# Supplementary material for: Electrolyte droplet spraying in H2 bubbles during water electrolysis under normal and microgravity conditions
Source: Nat Commun. 2025 May 16;16:4580. doi: 10.1038/s41467-025-59762-7 (PMC12084577; doi:10.1038/s41467-025-59762-7)
Supplement: Supplementary file 1 — Supplementary Information [file 41467_2025_59762_MOESM1_ESM.pdf]

Supplementary information: Electrolyte droplet spraying in H_2 bubbles during water electrolysis under normal and microgravity conditions

Aleksandr Bashkatov,[*,†,‡,¶] Florian Bürkle,[§] Çayan Demirkır,[‡] Wei Ding,[†] Vatsal Sanjay,[‡] Alexander Babich,[†] Xuegeng Yang,[†] Gerd Mutschke,[†] Jürgen Czarske,[§] Detlef Lohse,[‡,‖] Dominik Krug,[‡,¶] Lars Büttner,[§] and Kerstin Eckert[*,†,⊥]

†*Institute of Fluid Dynamics, Helmholtz-Zentrum Dresden-Rossendorf, Bautzner Landstrasse 400, 01328 Dresden, Germany*

‡*Physics of Fluids Group, Max Planck Center for Complex Fluid Dynamics and J. M. Burgers Centre for Fluid Dynamics, University of Twente, P.O. Box 217, 7500AE Enschede, Netherlands*

¶*Institute of Aerodynamics, RWTH Aachen University, Wüllnerstraße 5a, 52062 Aachen, Germany*

§*Laboratory for Measurement and Sensor System Techniques, Faculty of Electrical and Computer Engineering, Technische Universität Dresden, Helmholtzstr. 18, 01069 Dresden, Germany*

‖*Max Planck Institute for Dynamics and Self-Organization, Am Fassberg 17, 37077 Göttingen, Germany*

⊥*Institute of Process Engineering and Environmental Technology, Technische Universität Dresden, 01062 Dresden, Germany*

E-mail: a.bashkatov@hzdr.de; k.eckert@hzdr.de

Supplementary Note 1. Bubble dynamics in a microgravity environment

Supplementary Figure 1 demonstrates the evolution of H_2 bubble during water electrolysis at -4 V (vs. Pt wire) in 0.5 mol L^{-1} H_2SO_4 in a micro-g environment, i.e. at greatly eliminated buoyancy, achieved during the parabolic flights (see Bashkatov et al.[1]). The electric current in panel (a) reflects the dynamics of the bubble from its nucleation ($t = -14.71$ s) and until detachment ($t = 2.94$ s). The shadowgraphs in panel (b) depict the bubble position at the time instants marked by red circles in panel (a).

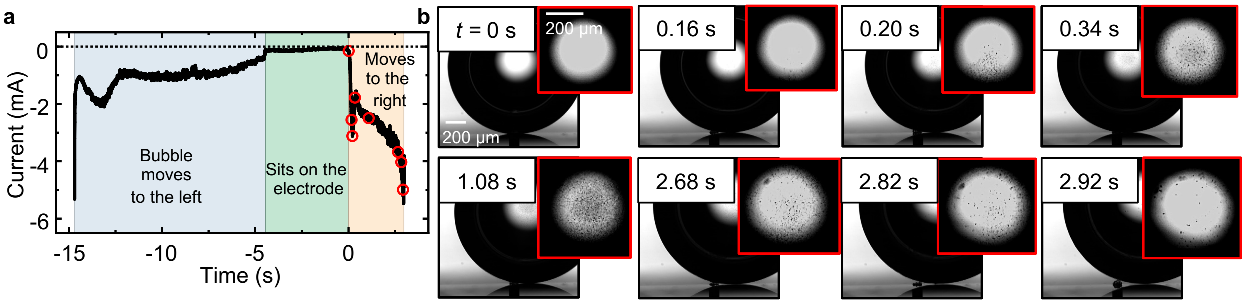

Supplementary Figure 1: **Electrolyte spraying in a microgravity environment.** The dynamics of H_2 bubble in micro-g achieved during parabolic flights: (a) electric current over the entire evolution cycle; (b) shadowgraphs of the bubble moving laterally (see Supplementary Movie 1) at the instants of time marked by the red circles in (a). The insets zoom into the central part of the bubble. Source data are provided as a Source Data file.

Here, due to a residual gravitation acceleration and its sign variation, the bubble evolution is characterized by the lateral motion shortly after its nucleation. During the first part of the evolution, it moves to the left (marked by grey shading in Suppl. Fig. 1a) relative to the electrode center. Later on, it rolls over the electrode, moving to the right while continuing to grow via coalescence with the carpet of microbubbles and diffusion. As the bubble rolls over the electrode, it blocks most of its active area, minimizing the electric current (marked by green shading in Suppl. Fig. 1a), hence electrochemical reaction and hydrogen production. The first snapshot ($t = 0$) marks the time instant shortly before the bubble releases the

electrode. The insets zoom into the central transparent part of the bubble. From the second snapshot onward, the bubble releases the electrode by moving to the right, drastically increasing the electric current (marked by yellow shading in Suppl. Fig. 1a) and allowing the formation of a carpet of microbubbles. The mother bubble continuously coalesces with newly nucleated bubbles (carpet) in the radial direction on a time scale of $\mathcal{O}(\mu s)$. The successive images reveal the internal flow of the microdroplets, emerging from the bottom, presumably at the coalescence spot, and flowing to the top, shortly after coalescence events begin. These droplets can be already seen in the second snapshot ($t = 0.16$ s). Since the bubble is displaced from the electrode center, the coalescence occurring in the radial direction results in the asymmetrical flow. Upon bubble departure, i.e., when no coalescence occurs, the already injected droplets exhibit minimal movement, drifting slowly due to residual velocity. For more details, we refer the readers to the manuscript.

Supplementary Note 2. Analytical aberration correction

Supplementary Figure 2a documents a schematic of the chief rays passing through the bubble surface. The solid lines mark the real path of the light scattered at the injected electrolyte droplets, whereas the dashed lines indicate the path recorded by the camera. Supplementary Figure 2b demonstrates a single ray passing through the bubble-electrolyte interface with the relevant geometry used in the calculations of the corrected position for each detected droplet. Since the bubble is assumed to be axisymmetric, the position of the droplet is defined by the radial distance from the bubble center in the plane of the laser sheet. The corrected position of each detected droplet can be calculated using Equation 2 (see manuscript).

Supplementary Figure 3 represents the effect of the correction, with panel (a) showing a raw snapshot of a H_2 bubble and the electrolyte droplets inside and panel (b) showing the same snapshot after performing the correction. In the raw image, the droplets are concentrated in the central part of the bubble, away from the interface. This is due to the

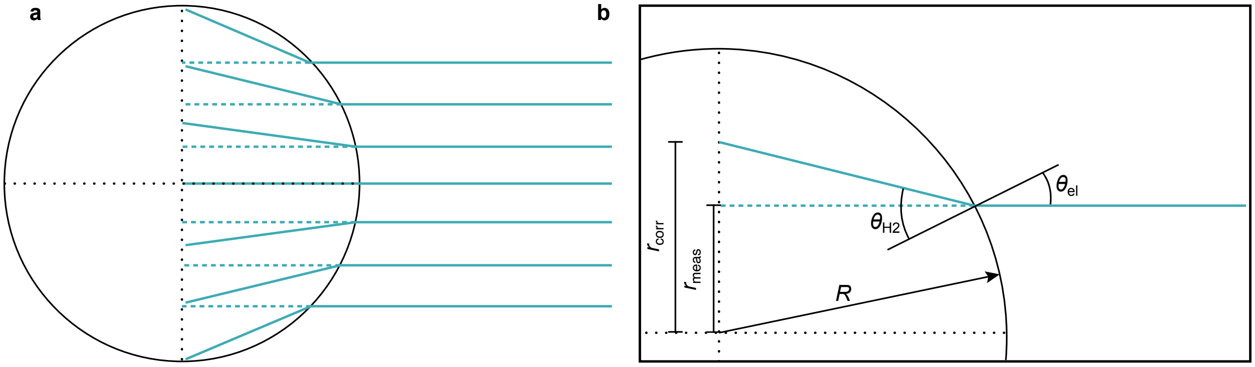

Supplementary Figure 2: **Optical ray path and distortion correction.** (a) Scheme of the chief rays in an object-space telecentric setup. The microscope objective is on the right side. The solid lines (cyan color) mark the real path of the light, whereas the dashed lines indicate the position in the image plane recorded by the camera. (b) A close view of a single ray passing through the bubble-electrolyte interface with the relevant geometry used in calculating the corrected position. r_{meas} and r_{corr} are the measured and corrected positions of the injected droplet, respectively. θ_{el} is the incident angle in the electrolyte, and θ_{H2} is the refracted angle inside the H_2 bubble.

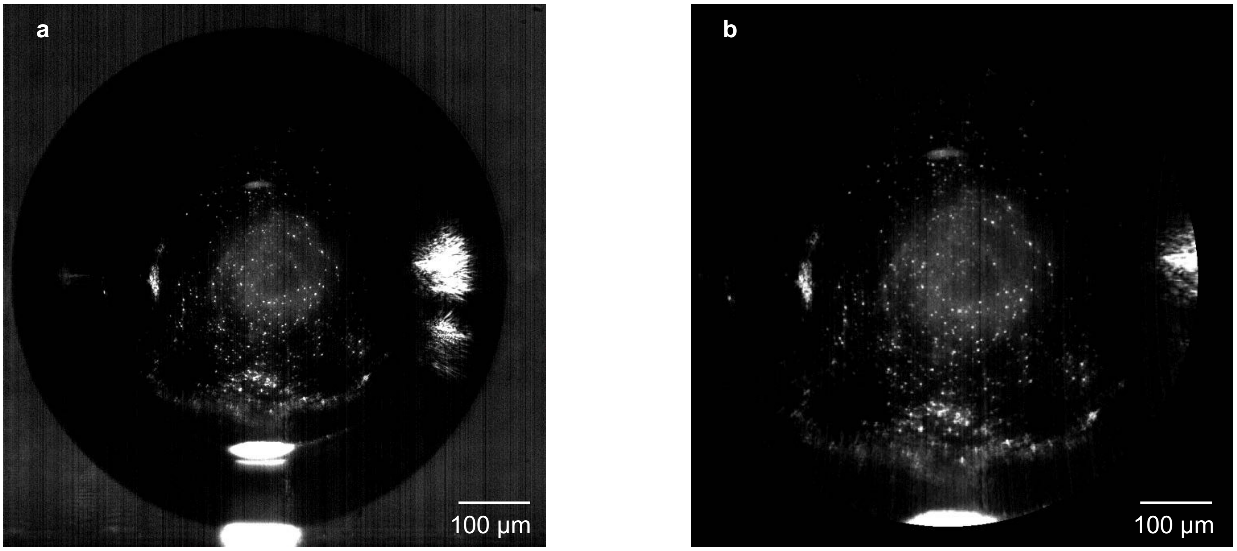

Supplementary Figure 3: **Droplet position correction.** (a) Original snapshot recorded by the camera. (b) Image representing the corrected position of the droplets. The pixels beyond the bubble interface are set to zero (black) in (b).

curvature of the bubble, which distorts the path of the rays. This is especially visible at the bottom of the bubble. The light scattered by the microbubble carpet (see manuscript) reaches the camera sensor both directly through the electrolyte and through the bubble. The light traveling through the bubble seems to come from a position closer to the center. When the correction is applied, the light traveling through the bubble seems to come from the bubble surface, which implies that the analytical model is correct (Suppl. Fig. 3b). Note that the model assumes a spherical shape of the droplet as an approximation. Deviations from the spherical shape of the refracting surface caused, e.g., by capillary waves or during detachment can potentially be corrected by approaches using adaptive optics.[2–4]

Supplementary Note 3. Bubble-carpet coalescence prior to departure

Supplementary Figure 4a documents the H_2 bubble shortly before its departure in 0.1 mol L^{-1} H_2SO_4 at −2.8 V (vs. RHE). The bubble resides above the electrode surface at the carpet of microbubbles having thickness δ and continuously coalesces with it.[5,6] Supplementary Figure 4b-e zooms in on the central segment of the bubble marked by the red rectangle in (a). The image recording was performed at 600 kHz and 720 kHz in (b-d) and (e), respectively.

The high-speed recording demonstrates that some of the droplets are injected at velocities three orders of magnitude larger than a majority of the droplets, reaching up to 15.8 m s^{-1}. These events are rather rare and can be observed only before and at the bubble departure, i.e., when the thickness of the carpet is maximum, about $\delta = 16$ µm and above. The larger δ will also result in droplets of bigger size.

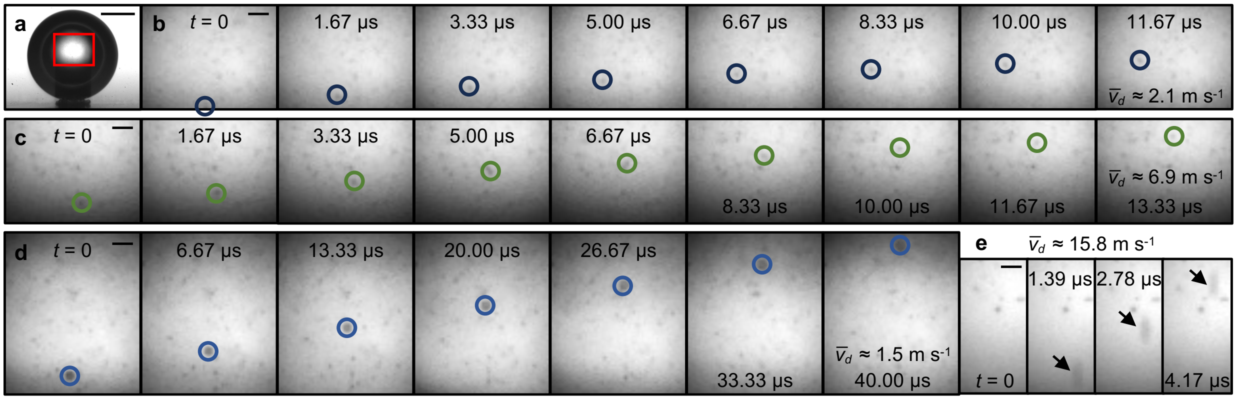

Supplementary Figure 4: **Electrolyte droplet dynamics before bubble departure.** (a) H_2 bubble growing at the electrode at −2.8 V (vs. RHE). (b-e) Zoom-in on the central segment of the bubble shown by the red rectangle in (a), demonstrating the motion of the injected electrolyte droplets, marked by black, green, or blue circles, with the velocity $\overline{v}_\mathrm{d}$. The image recording was performed at 600 kHz and 720 kHz in (b-d) and (e), respectively. The scale bar is 100 µm in (a) and 10 µm in (b-e). For further details, we refer to Supplementary Movies 8 to 11.

Supplementary Note 4. Stokes's drag: droplet velocity over time upon injection

The model experiment (see Fig. 4 in the manuscript) demonstrated that the coalescence of two unequal-sized bubbles is followed by an upward liquid jet, known as the Worthington jet, propagating inside of the merging H_2 bubbles. The jet will eventually break into droplet(s) due to either end-pinching or Rayleigh–Plateau instability moving with the velocities of m s^{-1} order of magnitude.

In the Stokes regime ($\mathrm{Re} < 1$), the injected droplet would experience drag (frictional) force and decelerate quickly due to viscous drag within the surrounding H_2 gas. For reference, the Reynolds number given as $\mathrm{Re} = (\rho_\mathrm{H2} \cdot v_\mathrm{d} \cdot 2R_\mathrm{d})/\mu_\mathrm{H2}$ equals to 0.5 with the radius of the droplet $R_\mathrm{d} = 5$ µm and velocity of the droplet $v_\mathrm{d} = 5$ m s^{-1}. ρ_H2 and μ_H2 are the density and dynamic viscosity of the hydrogen. We apply Newton's second law to the drag force,

$$m_\mathrm{d} \cdot \frac{dv_\mathrm{d}}{dt} = F_\mathrm{drag} = -6 \cdot \pi \cdot \mu_\mathrm{H2} \cdot R_\mathrm{d} \cdot v_\mathrm{d}, \tag{1}$$

where $m_{\rm d}$ is the mass of the droplet. By integrating Eq. 1, the velocity of the droplet over time reads

$$v_{\rm d}(t) = v_0 \cdot \exp\left(-t/\tau_{\rm d}\right), \tag{2}$$

where $\tau_{\rm d} = \frac{m_{\rm d}}{6 \cdot \pi \cdot \mu_{\rm H2} \cdot R_{\rm d}}$ and v_0 is an initial droplet velocity at the separation from the jet. Typically, $\tau_{\rm d} \approx 25$ µs ($R_{\rm d} = 1$ µm) and $\tau_{\rm d} \approx 650$ µs ($R_{\rm d} = 5$ µm), so drag quickly brings the flying drop to a stand-still.

Supplementary Figure 5 demonstrates (a) the velocity $v_{\rm d}(t)$ and (b) the traveled distance $S_{\rm d}(t) = \int_0^t v_{\rm d}(t')\,dt'$ of the injected droplet over time at three various sizes of $R_{\rm d} = 1$ µm, $R_{\rm d} = 3$ µm, and $R_{\rm d} = 5$ µm, plotted in black, red, and blue colors, respectively and with the initial velocities of $v_0 = 0.5$ m s^{-1} and $v_0 = 5$ m s^{-1} at $t_0 = 0$, plotted as solid and dotted lines, respectively. $S_{\rm d}(t)$ is calculated by integrating the $v_{\rm d}(t)$ in Supplementary Figure 5a. Both plots are presented on a semi-logarithmic scale.

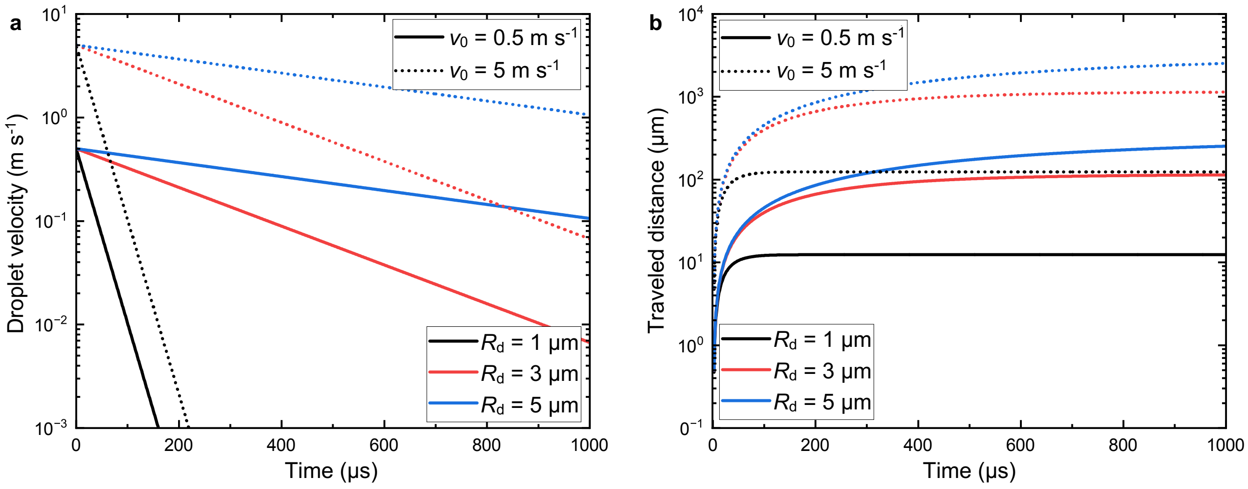

Supplementary Figure 5: **Temporal evolution of droplet motion.** (a) Velocity ($v_{\rm d}$) and (b) traveled distance ($S_{\rm d}$) of the injected droplet over time. Source data are provided as a Source Data file.

Upon the injection, the droplet velocity thus decays exponentially with time. The droplet

with $R_\mathrm{d} = 1$ µm (implies $\tau_\mathrm{d} \approx 25$ µs) and with $v_0 = 5$ m s^{-1} slows down to the velocity of 10^{-2} m s^{-1} at $t = 160$ µs by travelling $S_\mathrm{d} = 124$ µm. In comparison, by reducing the initial injection velocity (v_0), the droplet will reach the same order of magnitude (10^{-2} m s^{-1}) at $t = 100$ µs by traveling only $S_\mathrm{d} = 12$ µm. By increasing the droplet size from $R_\mathrm{d} = 1$ µm to $R_\mathrm{d} = 3$ µm and $R_\mathrm{d} = 5$ µm, the effect of viscous drag progressively reduces, so that the droplet at $v_0 = 5$ m s^{-1} can cover distances larger than 1 mm before the final position is reached (see Supplementary Figure 5b).

The droplet velocity v_d of 10^{-2} mm s^{-1} order of magnitude approximately resembles the one found in Fig. 2 (manuscript) at the bubble bottom in the bubble-carpet system.

Although it is not easy to determine the time of injection, since continuous coalescence events of the bubble carpet occur shortly after the formation of a single bubble on the electrode surface. We assume that the velocity is resolved at a distance between the aforementioned cases of $S_\mathrm{d} = 12$ µm and $S_\mathrm{d} = 100$ µm. away from the injection area. Rather, it is closer to $S_\mathrm{d} = 12$ µm, leading us to assume that the initial velocity is approximately $v_0 = 0.5$ m s^{-1}. This approximately resembles the velocity of the droplets ($R_\mathrm{d} = 1$ µm) in Fig. 2 (manuscript) resolved at about the same distance away from the injection cross-section at the bubble bottom in the bubble-carpet system.

Besides the initial conditions such as the size ratio of two coalescing bubbles and distorted symmetry of the coalescence as in the case of the bubble-carpet system (Figs. 1 and 2 in the manuscript), the initial velocity of the droplet will depend on which moment the droplet separates from the jet, i.e. whether it is the first or second droplet as in case of Fig. 4 (see manuscript). The last separation happened at the retraction of the jet, and therefore with a much smaller velocity, at 1.1 m s^{-1} and $R_\mathrm{d} = 22$ µm for the second droplet vs. 7.2 m s^{-1} and $R_\mathrm{d} = 13$ µm for the first droplet. The solution is not trivial since the first droplets are of smaller size than the last ones, and therefore slow down much faster, however, separating at a much faster initial velocity v_0.

In the case of the surface-attached bubble (Fig. 3, manuscript), the injected droplet

slows down upon the injection due to the Stokes's drag and then falls at the bubble-electrode contact area due to gravitational forces, presumably with the terminal velocity. In the Stokes regime (Re < 1), the terminal velocity of the droplet can be written

$$v_\mathrm{t} = \frac{2}{9}\frac{R_\mathrm{d}^2 \Delta\rho g}{\mu_\mathrm{H2}}, \tag{3}$$

where $\Delta\rho = \rho_\mathrm{el} - \rho_\mathrm{H2}$ is the electrolyte-gas density difference and μ_H2 the dynamic viscosity of the hydrogen. Given that the second observed droplet has a radius of about $r = 4$ μm, the terminal velocity $v_\mathrm{t} = 4.1$ mm s^{-1}. This value closely correlates with the experimentally estimated velocity of $\overline{v}_\mathrm{d} = 6$ mm s^{-1} during the period from $t = 1.33$ ms after injection until the droplet reaches the bubble-electrode contact area at $t = 86.67$ ms.

Supplementary Note 5. Mechanism of injection: experiment vs. simulation

The direct numerical simulation accurately reproduces the key features and timescales of phenomena such as neck formation, capillary wave propagation, jet formation, and droplet breakup. We then proceed to compare the details of drop ejection between the experiments and simulations presented in the manuscript body in Fig. 4. In the experiments, the first droplet with a radius of $R_\mathrm{d} = 13$ μm is observed at $t = 250.0$ μs and ejects with a velocity of approximately 7.2 m s^{-1}. The second droplet, with a radius of $R_\mathrm{d} = 22$ μm, appears at $t = 341.7$ μs and separates just before the jet starts to retract between $t = 333.3$ μs and $t = 341.7$ μs, resulting in a much smaller velocity of about 1.1 m s^{-1}. In contrast, in the simulations, the first droplet (radius $R_\mathrm{d} = 15$ μm) pinches off at $t = 260$ μs with a velocity of $\overline{v}_\mathrm{d} = 4.3$ m s^{-1}. The second droplet (radius $R_\mathrm{d} = 18$ μm) pinches off at $t = 285$ μs with a velocity of $\overline{v}_\mathrm{d} = 3.4$ m s^{-1}. Additionally, another droplet (radius $R_\mathrm{d} = 3$ μm) was detected

at an earlier phase ($t = 208\,\mu s$) with a much faster velocity of $\overline{v}_\mathrm{d} = 43\,\mathrm{m\ s^{-1}}$, likely due to numerical artifacts caused by finite mesh resolution. This droplet can be barely seen in Fig. 4 (see manuscript) at $t = 208$ µs. The characteristics of the injected droplets are summarized in Table 1.

Supplementary Table 1: Comparison of the droplet injection characteristics between experiment and simulations. t represents the time from the onset of coalescence, R_d and $\overline{v}_\mathrm{d}$ the radius and the velocity of the injected droplet, respectively. In the experiment, only two droplets (#1 and #2) are observed, whereas the simulation shows three droplets (#0, #1, and #2). Droplet #0 is likely resolved due to numerical artifacts caused by finite mesh resolution.

Droplet (#)	t (µs)		R_d (µm)		$\overline{v}_\mathrm{d}$ (m s^{-1})	
	experiment	simulation	experiment	simulation	experiment	simulation
0	-	208	-	3	-	43
1	250	260	13	15	7.2	4.3
2	341.7	285	22	18	1.1	3.4

Supplementary references

(1) Bashkatov, A.; Yang, X.; Mutschke, G.; Fritzsche, B.; Hossain, S. S.; Eckert, K. Dynamics of single hydrogen bubbles at Pt microelectrodes in microgravity. *Phys. Chem. Chem. Phys.* **2021**, *23*, 11818–11830.

(2) Bilsing, C.; Nützenadel, E.; Burgmann, S.; Czarske, J.; Büttner, L. Adaptive-optical 3D microscopy for microfluidic multiphase flows. *Light: Adv. Manuf.* **2024**, *5*, 385–399.

(3) Gao, Z.; Radner, H.; Büttner, L.; Ye, H.; Li, X.; Czarske, J. Distortion correction for particle image velocimetry using multiple-input deep convolutional neural network and Hartmann-Shack sensing. *Opt. Express* **2021**, *29*, 18669–18687.

(4) Radner, H.; Stange, J.; Büttner, L.; Czarske, J. Field-programmable system-on-chip-based control system for real-time distortion correction in optical imaging. *IEEE Trans. Ind. Electron.* **2020**, *68*, 3370–3379.

(5) Bashkatov, A.; Hossain, S. S.; Yang, X.; Mutschke, G.; Eckert, K. Oscillating hydrogen bubbles at Pt microelectrodes. *Phys. Rev. Lett.* **2019**, *123*, 214503.

(6) Bashkatov, A.; Hossain, S. S.; Mutschke, G.; Yang, X.; Rox, H.; Weidinger, I. M.; Eckert, K. On the growth regimes of hydrogen bubbles at microelectrodes. *Phys. Chem. Chem. Phys.* **2022**, *24*, 26738–26752.
