# Supplementary material for: Electrolyte droplet spraying in H2 bubbles during water electrolysis under normal and microgravity conditions
Source: Nat Commun. 2025 May 16;16:4580. doi: 10.1038/s41467-025-59762-7 (PMC12084577; doi:10.1038/s41467-025-59762-7)
Supplement: Supplementary file 2 — Description of Additional Supplementary Files [file 41467_2025_59762_MOESM2_ESM.pdf]

Description of Additional Supplementary Files

File Name: Supplementary Movie 1

Description: (Figure 1) Movie demonstrating the stream of electrolyte microdroplets inside an H_2 bubble during the late phase of its evolution in a micro-*g* environment at -4 V (vs. Pt wire) in 0.5 M H_2SO_4.

File Name: Supplementary Movie 2

Description: (Figure 2a) Movie demonstrating the bubble evolution at the micro-electrode and corresponding droplets injection as a result of bubble-carpet coalescence at -3 V (vs. Ag/AgCl) in 0.1 M H_2SO_4.

File Name: Supplementary Movie 3

Description: (Figure 2b) Result of Particle Tracking Velocimetry (PTV) measurements demonstrating electrolyte spraying within an H_2 bubble at -3 V (vs. Ag/AgCl) in 0.1 M H_2SO_4.

File Name: Supplementary Movie 4

Description: (Figure 2c) Result of Particle Tracking Velocimetry (PTV) measurements demonstrating electrolyte spraying within an H_2 bubble at -7 V (vs. Ag/AgCl) in 0.1 M H_2SO_4.

File Name: Supplementary Movie 5

Description: (Figure 3ab) Movie focusing on the contact patch of the electrode-attached bubble and demonstrating the development of the electrolyte puddles throughout of the bubble evolution at -50 A m^{-2} in 0.1 M $HClO_4$. The experiments were performed from underneath the electrode (transparent) of the growing H_2 bubble.

File Name: Supplementary Movie 6

Description: (Figure 3d) Movie focusing on the contact patch of the electrode-attached bubble and illustrating the injection of microdroplets upon coalescence event followed by their sedimentation at the contact area (-50 A m^{-2} in 0.1 M $HClO_4$).

File Name: Supplementary Movie 7

Description: (Figure 4a) Droplet ejection mechanism upon coalescence of two unequal size H_2 bubbles (R_b = 400 μm and R_s = 205 μm). Image recording was performed at 120 kHz.

File Name: Supplementary Movie 8

Description: (Supplementary Fig. 4b) Zoom-in on the central segment of the bubble (see Figure 4a in Supplementary Information), demonstrating the motion of the injected electrolyte droplets. The image recording was performed at 600 kHz. The video is slowed down 120000 times. The playback speed is 5 frames per second (total 19 frames). The spatial resolution is 996 pix mm^{-1}. The frame width is 64 pixels and the height is 128 pixels.

File Name: Supplementary Movie 9

Description: (Supplementary Fig. 4c) Zoom-in on the central segment of the bubble (see Figure 4a in Supplementary Information), demonstrating the motion of the injected electrolyte droplets. The image recording was performed at 600 kHz. The video is slowed down 120000 times. The playback speed is 5 frames per second (total 18 frames). The spatial resolution is 996 pix mm^{-1}. The frame width is 64 pixels and the height is 128 pixels.

File Name: Supplementary Movie 10

Description: (Supplementary Fig. 4d) Zoom-in on the central segment of the bubble (see Figure 4a in Supplementary Information), demonstrating the motion of the injected electrolyte droplets. The image recording was performed at 600 kHz. The video is slowed down 120000 times. The playback speed is 5 frames per second (total 35 frames). The spatial resolution is 996 pix mm^{-1}. The frame width is 64 pixels and the height is 128 pixels.

File Name: Supplementary Movie 11

Description: (Supplementary Fig. 4e) Zoom-in on the central segment of the bubble (see Figure 4a in Supplementary Information), demonstrating the motion of the injected electrolyte droplets. The image recording was performed at 720 kHz. The video is slowed down 144000 times. The playback speed is 5 frames per second (total 25 frames). The spatial resolution is 996 pix mm^{-1}. The frame width is 32 pixels and the height is 128 pixels.
